# Numerical Investigation of Nanostructure Orientation on Electroosmotic Flow

**DOI:** 10.3390/mi11110971

**Published:** 2020-10-29

**Authors:** An Eng Lim, Yee Cheong Lam

**Affiliations:** School of Mechanical and Aerospace Engineering, Nanyang Technological University, 50 Nanyang Avenue, Singapore 639798, Singapore; lima0028@e.ntu.edu.sg

**Keywords:** electrokinetic phenomenon, electroosmotic flow, micro-/nanofluidics, nanoline nanostructures, numerical simulation

## Abstract

Electroosmotic flow (EOF) is fluid flow induced by an applied electric field, which has been widely employed in various micro-/nanofluidic applications. Past investigations have revealed that the presence of nanostructures in microchannel reduces EOF. Hitherto, the angle-dependent behavior of nanoline structures on EOF has not yet been studied in detail and its understanding is lacking. Numerical analyses of the effect of nanoline orientation angle *θ* on EOF to reveal the associated mechanisms were conducted in this investigation. When *θ* increases from 5° to 90° (from parallel to perpendicular to the flow direction), the average EOF velocity decreases exponentially due to the increase in distortion of the applied electric field distribution at the structured surface, as a result of the increased apparent nanolines per unit microchannel length. With increasing nanoline width *W*, the decrease of average EOF velocity is fairly linear, attributed to the simultaneous narrowing of nanoline ridge (high local fluid velocity region). While increasing nanoline depth *D* results in a monotonic decrease of the average EOF velocity. This reduction stabilizes for aspect ratio *D*/*W* > 0.5 as the electric field distribution distortion within the nanoline trench remains nearly constant. This investigation reveals that the effects on EOF of nanolines, and by extrapolation for any nanostructures, may be directly attributed to their effects on the distortion of the applied electric field distribution within a microchannel.

## 1. Introduction

Electroosmotic flow (EOF) in a porous material or micro-/nanodimensional channel is an electric-field-induced fluid flow, and is an electrokinetic phenomenon. When a solid surface is exposed to an aqueous solution, the developed negative charges next to the channel wall attract and repel positive and negative ions, respectively. This leads to the formation of a net positively charged layer known as the electrical double layer (EDL) of nanometer thickness. Upon the application of an electric field parallel to the channel surface, the EDL experiences an electric body force that drives its motion in the field direction. Across the channel, a pluglike flow is generated as a result of the transfer of momentum from the EDL to the bulk fluid through viscous drag. When the EDL thickness is thin compared to the dimensions of the fluidic channel, the EOF velocity may be calculated by the Helmholtz–Smoluchowski equation:

*V_EOF_* = −*ɛ_r_ɛ_o_ζE*/*µ*, (1)

where *E* is the external applied electric field, *ε_o_* is the permittivity of free space, *ε_r_* is the relative permittivity of liquid, *µ* is the liquid viscosity, and *ζ* is the zeta potential (electrostatic potential developed on the channel surface). The Debye length characterizes the thickness of EDL (for a symmetric electrolyte):

*λ_D_* = (*ɛ_r_ɛ_o_k_b_T*/2*z*^2^*e*^2^*N_a_c_o_*)^1/2^, (2)

where *e* is the electron charge, *z* is the absolute charge of ion species, *c_o_* is the solution concentration, *N_a_* is the Avogadro constant, *k_b_* is the Boltzmann constant, and *T* is the temperature.

EOF has been extensively employed in a vast range of applications, such as remediation of contaminated soils [1], transportation of dental composites [2], liquid chromatography [3], multiphase electrokinetic flow [4,5], electrokinetically controlled DNA hybridization [6,7], and controlled drug release [8,9,10]. Multiphase flow [11,12] has been promising for enhancing the efficiency of numerous processes, such as chemical and biocatalytic reactions [13,14]. The application of EOF in a multiphase system serves to manipulate (i.e., form, sort, or merge) the different phases on demand [4,5] which is crucial for desirable experimental outcomes. One of the main problems of DNA microarray hybridization is the long turnaround time and low sensitivity due to diffusion-limited reaction kinetics [15]. Differing from the conventional techniques such as acoustic wave agitation [16] and magnetic stirring [17] that promote fluid mixing and mass transfer, electrokinetic transport method [6,7] offers more precise fluidic control, ease of operation, flexibility for miniaturization, and fabrication simplicity. Electrokinetic delivery also has great potential in micro-electro-mechanical system (MEMS) devices for controlled drug release applications, which often employ sophisticated channel designs such as microchannels with embedded magnetic nanoparticles for effective drug delivery [8,9,10].

Despite the broad applicability of EOF, there exists significant discrepancies between classical theory and experimental observations. For instance, induced-charge electroosmotic flow (ICEOF) [18] at a large applied voltage results in high electric field and induced zeta potential, which lead to deviations from the theoretical predictions because of ion steric effects [19], ion–ion couplings [20], viscoelectric effects [21], and Faradaic reactions [22]. It has been demonstrated that EOF between two fluids with dissimilar concentrations [23,24] and ionic species [25,26] exhibits hysteretic behavior (known as EOF hysteresis), whereby the flow behavior differs depending on the flow direction. Contrary to the prediction of prevailing EOF theory, EOF hysteresis originates from the depletion/accumulation of the minority pH-governing ion concentrations [23,24] and the concentration adjustment of the major constituent ion species [25,26], which alters the zeta potentials, and thus the fluid flow velocities for different flow directions.

Surface roughness, which is inevitably formed in micro-/nanofluidic devices as a result of the manufacturing process [27,28], can also significantly influence the EOF velocity. Several simulation studies have been conducted to investigate the effects of surface roughness on EOF [29,30,31,32,33,34,35,36]. Hu et al. [29,30,31] studied the effect of microscale roughness on EOF in a microchannel with square prismatic elements. They demonstrated that the presence of roughness elements in the flow passage induces a periodic pressure field, which causes nonuniformity of the fluid velocity that greatly reduces the EOF flow rate. Kang and Suh [32] and Masilamani et al. [33] investigated EOF in a microchannel with microscale, rectangle-waved surface roughness. They discovered that fluid flows along the waved wall surface without flow separation, and the EOF flow rate increases with the periodic length and decreases with the amplitude of the waved wall.

Kim and Darve [34] performed a simulation study to examine the effect of subnanoscale grooves and ridges (rectangle-waved surface roughness) on EOF in nanochannel. The EOF velocity profiles correspond to those with overlapping EDLs [37,38,39], and the flow rate was found to increase with increasing period and decrease with increasing amplitude of surface roughness. This coincides with the investigations carried out by Kang and Suh [32] and Masilamani et al. [33]. Messinger and Squires [35] revealed that nanoscale surface curvatures, which typically form during the microfabrication of metal electrodes, can significantly lower the EOF velocity when there is appreciable excess surface conductivity within the EDL. Kaji et al. [36] confirmed through simulations that the presence of nanoscale pillarlike structures in a microchannel suppresses EOF, which enhances the resolution of DNA separation.

Though there is an abundance of simulation studies, limited experimental investigations have been conducted [40,41,42,43] due to fabrication challenges, especially large-area nanoscale surface patterning, which is costly and technically difficult to control. Koga et al. [40] investigated the effect of random surface roughness (in scale of nanometers) on EOF with polydimethylsiloxane (PDMS)-glass hybrid microchannels. The glass surfaces were treated with dry and wet etchings, i.e., neutral loop discharge (NLD) plasma and hydrofluoric acid (HF) etchings, for the alteration of the average arithmetic roughness *R_a_*. Their investigation revealed that EOF velocity has a strong correlation with *R_a_*. The surfaces with high *R_a_* (achieved with dry etching) show a notable reduction in the EOF velocity as compared to the surfaces with low *R_a_* (achieved with wet etching).

In view of the employment of etching process for large-area surface structuring by Koga et al. [40], our group proposed the DEEMO (Dry Etching, Electroplating, and MOlding) process for the fabrication of microfluidic channel with black silicon nanostructures (prolate hemispheroid-like structures, which are moderately regular) to investigate its effect on EOF [41]. The black silicon nanostructures were generated by reactive ion etching (RIE), whereby the passivating (O_2_) and etching (CH_4_ and/or SF_6_) gas flow rates can be tuned to produce different nanostructure dimensions. There was a considerable decrease in the average EOF velocity when the black silicon nanostructures were introduced in the microchannel.

Yasui et al. [42] adopted electron beam lithography (EBL), which is a precise nanopatterning technique, for the fabrication of nanopillar arrays in microchannels to examine its effect on EOF. It was discovered that the presence of nanopillar arrays has an inherent ability for EOF suppression, and the flow velocity is affected by the total number of nanopillars inside the microchannel. They also reported that the different arrangements of nanopillars in square or tilted arrays have no observable difference in EOF velocity. This could well be attributed to the minimal orientation difference between these arrangements.

Recently, our group developed a novel technique [43], namely, a hybrid LIGA-DEEMO process (LIthographie, Galvanoformung, and Abformung; which stands for Lithography, Electroplating, and Molding in German), to fabricate microchannels with nanostructure designs that possess extreme orientation difference, i.e., perpendicular and parallel nanolines. Our recent investigation [43] demonstrated conclusively that the perpendicular nanolines significantly reduce the EOF velocity. In contrast, the parallel nanolines have negligible effect on EOF. However, the angle-dependent behavior of the nanolines on EOF has not yet been fully investigated. Furthermore, there is a lack of detailed substantiation analysis by the existing literatures [29,30,31,32,33,34,35,36,40,41,42,43] on the mechanics of EOF reduction due to the presence of nanostructures in microchannel. 

A thorough numerical analysis of the nanoline orientation angle variation effect on EOF and the associated mechanisms was performed. In addition, the effects of varying the nanoline width and depth in conjunction with the variation of orientation angle were carried out. This investigation aims to deepen the understanding of EOF in microfluidic devices with patterned nanostructured surfaces to gain precise and accurate flow manipulation for applied analytical purposes in areas such as biochemistry and life science. 

## 2. Numerical Simulations

### 2.1. Simulation Domain

**Figure 1:**
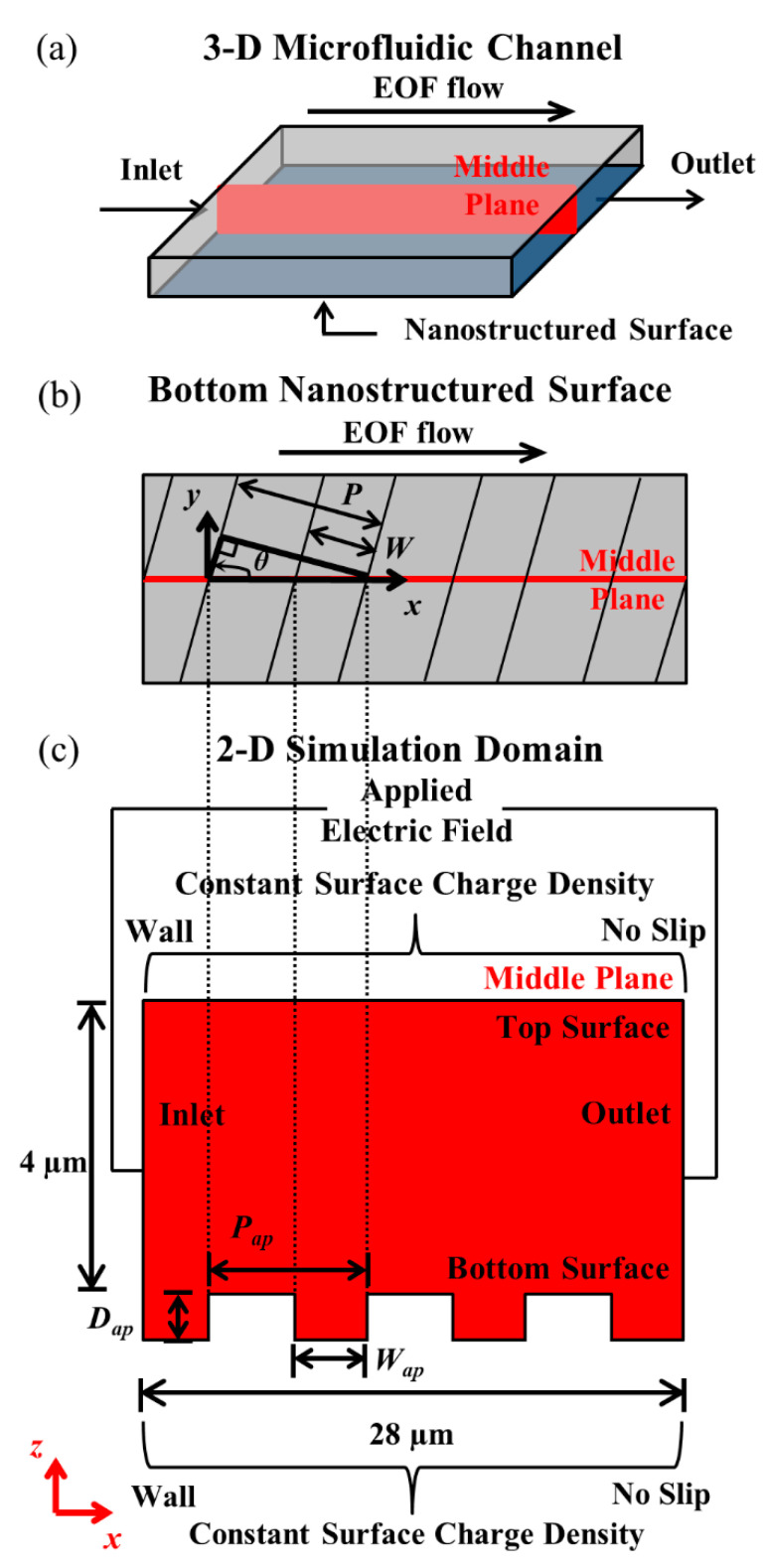
(**a**) Three-dimensional (3-D) microfluidic channel. (**b**) Bottom surface of 3-D microchannel with nanolines. (**c**) Two-dimensional (2-D) simulation domain (middle plane of 3-D microchannel). Figures are not drawn to scale. For nanolines, *θ* is orientation angle, *P* is period, *W* is width, *D* is depth; with *P_ap_* (= *P*/sin*θ*)*, W_ap_* (= *W*/sin*θ*), and *D_ap_* (= *D*) as apparent period, apparent width, and apparent depth, respectively; where 0 < *θ* ≤ 90°.

Numerical studies were conducted with finite element method (FEM), with the simulations implemented on COMSOL Multiphysics. Without sacrificing generality, and for ease of problem formulation, two-dimensional (2-D) simulations were employed. The simulation domain consists of the middle plane across a three-dimensional (3-D) rectangular microchannel, with nanolines of depth *D*, width *W*, and period *P* at the bottom surface of the microchannel (see Figure 1a,b). As shown in Figure 1b, the orientation angle *θ* of the nanolines is measured counter-clockwise with respect to the *x*-axis (direction of EOF). For the 2-D simulation of the middle plane, the apparent depth *D_ap_* is equal to the true depth *D* regardless of *θ*, while the apparent width *W_ap_* and period *P_ap_* of the nanolines can be derived geometrically (see Figure 1b,c) with Equation (3) and Equation (4), respectively:

*W_ap_* = *W/*sin*θ*, where 0 < *θ* ≤ 90°, (3)

*P_ap_* = *P/*sin*θ*, where 0 < *θ* ≤ 90°. (4)

In this numerical investigation, steady-state EOF in microchannels of various nanoline orientation angles—i.e., *θ* = 5°, 15°, 30°, 45°, 60°, 75°, and 90°—were simulated by Poisson–Nernst–Planck (PNP) model with modified boundary conditions [25,26,41,43]. The governing equations for the PNP model are discussed in Section 2.3. The effect of varying the nanoline orientation angle on EOF was investigated with different sodium bicarbonate (NaHCO_3_) concentrations, i.e., 0.25 mM, 0.50 mM, 1.0 mM, 2.0 mM, and 4.0 mM. For this investigation, the nanoline depth *D*, width *W*, and period *P* were fixed at 90 nm, 160 nm, and 400 nm, respectively; these chosen dimensions are close to our previous experimental investigation [43].

The Debye length for the NaHCO_3_ concentrations is at most in the order of tens of nanometers (i.e., 19.4 nm for 0.25 mM NaHCO_3_, as calculated by Equation (2)). In comparison, the nanoline width *W*, which is a critical dimension for trench formation, is in the order of hundreds of nanometers (i.e., *W* = 160 nm). Hence, localized ion polarization within the trench (resulting in nonuniform electric field strength around the trench top that reduces EOF) is unlikely to occur and need not be considered for the simulations.

From our previous experimental investigations [41,43], photolithography smooth microchannel surface has roughness arithmetic mean *Ra* = 5.5 ± 0.6 nm. As the *Ra* of the microchannel surface is in the nanometer range, with the Debye lengths (as calculated by Equation (2)) and nanoline structures in the order of tens and hundreds of nanometers, respectively; and channel features in the micrometer range, the lithography roughness could well be considered as smooth in comparison with the nanostructures and microchannels. Thus, the roughness has negligible contribution to EOF in processes for the microfluidic channels investigated.

In addition, with NaHCO_3_ concentration fixed at 1.0 mM, the effects of varying both *W* and *D* were investigated in conjunction with a variation of *θ*. With *P* and *D* fixed at 400 nm and 90 nm respectively, the values of *W* investigated were 80 nm, 160 nm, 240 nm, and 320 nm. With *W* and *P* fixed at 160 nm and 400 nm respectively, the values of *D* investigated were 22.5 nm, 45 nm, 90 nm, and 135 nm. It is to be highlighted here that for all these investigations, the EDL thickness for the different NaHCO_3_ concentrations characterized by the Debye length (see Equation (2)), are thin compared to the micro-/nanostructures. Hence, the EDL does not overlap. This ensures that the phenomena associated with overlapping EDL such as concentration polarization [44], extended space charge layers [45], and vortex formation [46] will not have an effect on the simulated EOF. 

The 2-D simulation domain (see Figure 1c) was meshed with triangular elements. To resolve the steep variable changes across the EDL, high element mesh density was employed near the vicinity of the microchannel wall boundaries. The maximum element size at the smooth/nanostructured wall boundary was prescribed to be 2.5 nm. The adjacent mesh size increased at a maximum element growth factor of 1.05 with increasing distance from the smooth/nanostructured wall boundary. Convergence test was conducted with smaller maximum element size prescribed at the wall boundary, i.e., 1.5 nm (40% reduction). It was found that the percentage changes in the results were less than 0.1%.

### 2.2. Boundary Conditions

The inlet of the simulation domain (see Figure 1c) was prescribed with a voltage of 0.28 V to establish an electric field of 100 V·cm**^−^**^1^. Perfect insulation condition was set at the smooth/nanostructured wall boundary, which is a good approximation for most applications with polymeric microfluidic devices as the substrate is an excellent insulator. In some special circumstances where the applications require channel substrates of nonperfect insulation materials, electric field leakages at the nanostructure corners can occur which may lead to vortexes that would further reduce EOF [47].

No specific absorption of ion on the wall surface was assumed [48] such that a constant value of wall surface charge can be specified for all NaHCO_3_ concentrations; this allows zeta potential variation as a function of the solution concentration. The wall surface charge density *S* was prescribed as −9.86 × 10^−^^3^ C·m^−2^, which is the experimental measured value for cyclic olefin copolymer (COC) material based on 1.0 mM NaHCO_3_ [43]. Electroneutrality condition was imposed on the domain, except on the smooth/nanostructured wall boundary. This allows EDL formation, which consists of a net positive charge for balancing the negative surface charge prescribed on the wall boundary. An electrical body force can then be generated in the EDL through the application of an electric field, which drives the bulk fluid movement by viscous effects.

For ion concentrations under the influence of electrostatic wall potential, Boltzmann distribution was assumed at the inlet/outlet of the domain. The pressure gradient normal to the wall boundary will be cancelled out by the electrostatic wall potential induced body force [49]. The pressure was set as zero for the inlet/outlet, and no slip condition was prescribed at the smooth/nanostructured wall. Detailed boundary conditions for the 2-D numerical simulation of a steady-state EOF is shown in Table 1.

### 2.3. Governing Equations for Poisson–Nernst–Planck (PNP) Model

The PNP model adopted here is as outlined in our previous investigations [25,26,41,43]. The development of the governing equations may be summarized as follows.

Laplace equation, which governs the applied electric potential *φ*, is essential for a proper description of the critical phenomenon associated with electric field variation:

∇·(*σ*∇*φ*) = 0, (5)

where *σ = F∑z_i_u_m(i)_c_i_* is the solution conductivity, *F* is the Faraday constant, *z_i_* is the charge number, *u_m(i)_* is the ionic mobility, and *c_i_* is the concentration of the ion species.

Poisson equation describes the electrostatic wall potential distribution *ψ* (due to the prescribed wall surface charge density):

∇·∇*ψ* = −*ρ_e_*/*ɛ_r_ɛ_o_*, (6)

where *ρ_e_* = *F*∑*c_i_z_i_* is the net charge density, *ε_r_* is the relative permittivity of fluid, and *ε_o_* is the permittivity of free space. By specifying a constant surface charge as the wall condition, the variation of zeta potential as a function of solution concentration can be described.

Nernst–Planck (NP) equation simulates the ion species distributions, which is governed by the gradients of diffusive, electromigrative, and convective fluxes, respectively:

∇·[−*D_i_*∇*c_i_* – *u_m(i)_c_i_*∇(*φ* + *ψ*)] + ***v***·∇*c_i_* = 0, (7)

where *D_i_* is the diffusion coefficient of ion species and *v* is the fluid velocity.

Navier–Stokes (NS) and continuity equations define the fluid flow for an incompressible Newtonian fluid:

−∇*p* + *µ*∇^2^***v*** + *ρ_e_*[−∇(*φ*)] = 0, (8)

∇·***v*** = 0, (9)

where *p* is the pressure, *µ* is the viscosity, and *ρ* is the density of the fluid. Due to the small channel dimensions in microfluidics, the Reynolds number is typically less than 1. As such, the inertial term was ignored, and Stokes flow was assumed. The electrostatic wall potential induced body force was omitted from the electrical body force term as it was presumed to be balanced by the pressure gradient normal to the channel wall [49].

The governing equations (see Equation (5) to Equation (9)) were solved simultaneously to generate the steady-state EOF. The pressure and velocity were discretized with linear elements, while the applied electric potential, electrostatic wall potential, and ion concentrations were discretized with quadratic elements. The convergence criterion was based on a relative tolerance of less than 0.001 between subsequent iterations, i.e., the computed state is accurate to approximately 0.1%. The symbols and values of constants used for the numerical simulations are contained in Table 2.

## 3. Results and Discussion

**Figure 2:**
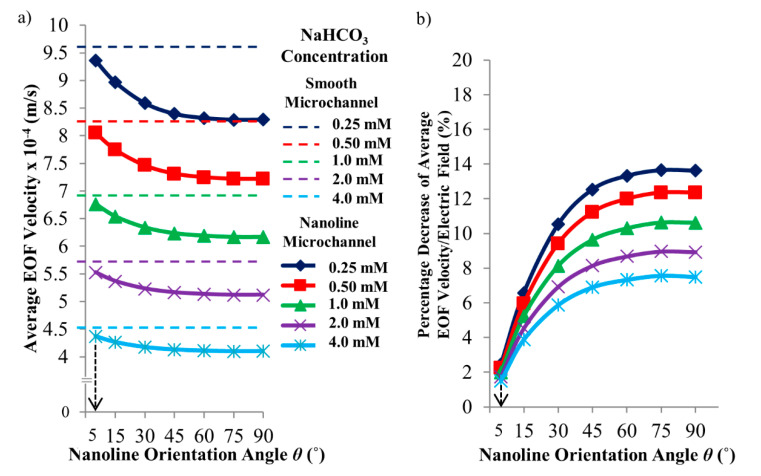
(**a**) Simulated average electroosmotic flow (EOF) velocity as a function of sodium bicarbonate (NaHCO_3_) concentration and nanoline orientation angle *θ*. (**b**) Percentage decrease of average EOF velocity, as well as average electric field strength, of nanoline microchannel compared to smooth channel. Nanoline width *W*, period *P*, and depth *D* were fixed at 160 nm, 400 nm, and 90 nm, respectively.

Figure 2a shows the simulated average EOF velocities of 0.25 mM, 0.50 mM, 1.0 mM, 2.0 mM, and 4.0 mM NaHCO_3_ concentrations in the microchannels with different nanoline orientation angles. The EOF velocities for various nanoline orientation angles decrease with increasing concentration of NaHCO_3_ (see Figure 2a). The thickness of EDL (which is characterized by the Debye length, see Equation (2)) decreases when the NaHCO_3_ concentration is increased. With increasing solution concentration, there is a higher concentration of ion species presents for the screening of the wall surface charge, which leads to the decrease of the EDL thickness. This in turn results in a lower zeta potential value that causes the decrease in the EOF velocity (see Equation (1)) when the concentration of NaHCO_3_ is increased. 

Figure 2b shows the percentage decrease of the average EOF velocity, as well as the average electric field strength, of a nanoline microchannel as compared to a smooth channel for the same NaHCO_3_ concentrations. Due to the variation of the nanoline orientation angle *θ* for different NaHCO_3_ concentrations, the percentage decrease of the average EOF velocity follows a similar trend, i.e., it increases with an increase in *θ* (see Figure 2b). The percentage decrease of the EOF velocity increases with decreasing NaHCO_3_ concentration. For instance, where 5° ≤ *θ* ≤ 90°, the percentage decrease of the EOF velocity ranges between 2.44% to 13.6% for 0.25 mM NaHCO_3_, and 1.50% to 7.49% for 4.0 mM NaHCO_3_ (see Figure 2b). According to Equation (1), the EOF velocity varies directly with the electric field, which is shown in Figure 2b. The decrease of the average EOF velocity occurs as a result of the average electric field reduction due to the distortion of the local electric field distribution at the wall surface, with the presence of nanolines at various *θ*.

**Figure 3:**
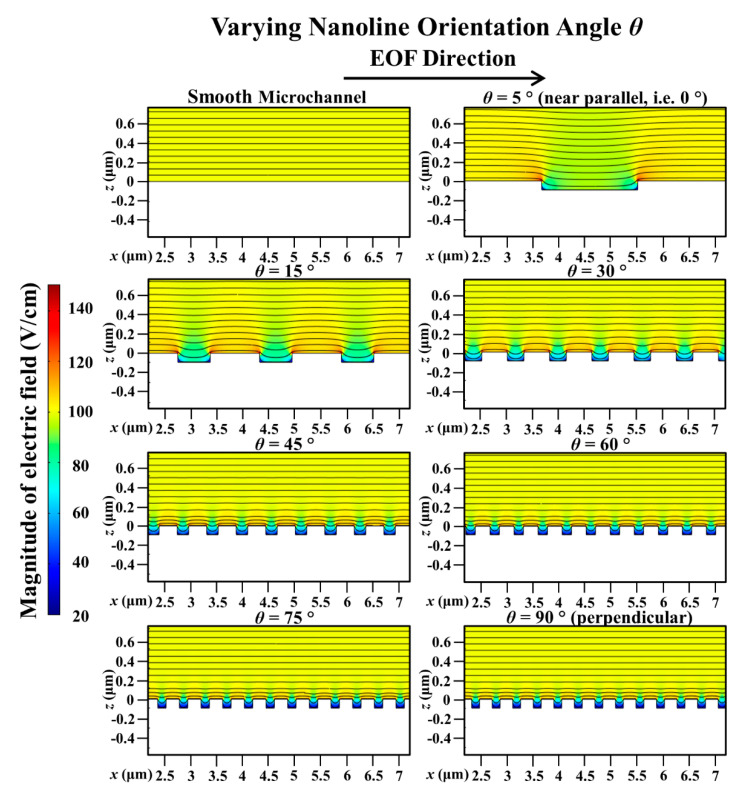
Simulated electric field distribution in microchannel with varying nanoline orientation angle *θ*, in comparison to smooth microchannel. Sodium bicarbonate (NaHCO_3_) concentration was fixed at 1.0 mM, and nanoline width *W*, period *P*, and depth *D* were fixed at 160 nm, 400 nm, and 90 nm, respectively. When *θ* = 5°, distortion of electric field distribution is less severe. As *θ* increases, distortion increases due to increased number of apparent nanolines.

**Figure 4:**
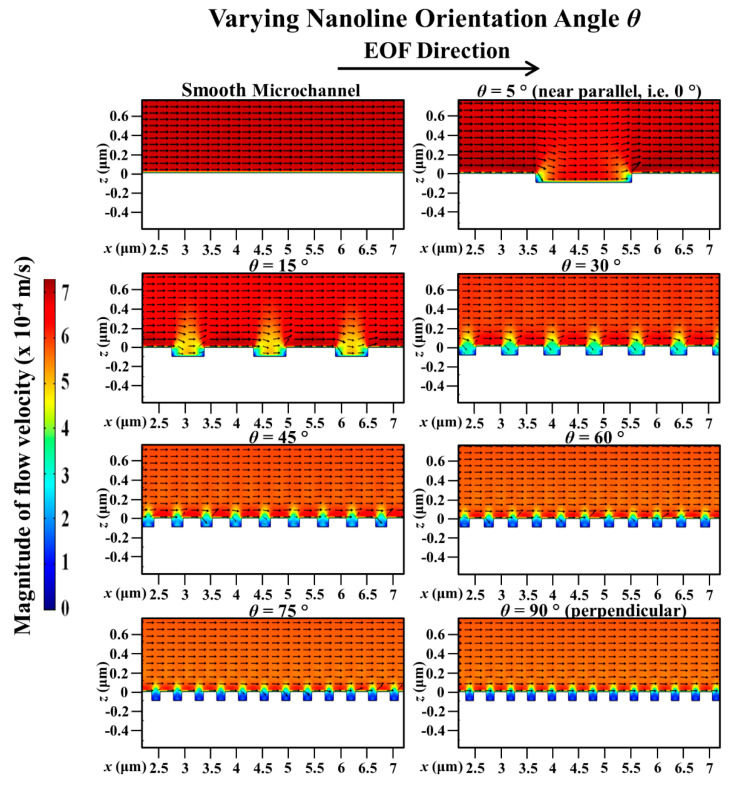
Simulated electroosmotic flow (EOF) velocity profile in microchannel with varying nanoline orientation angle *θ*, in comparison to smooth microchannel. Sodium bicarbonate (NaHCO_3_) concentration was fixed at 1.0 mM, and nanoline width *W*, period *P*, and depth *D* were fixed at 160 nm, 400 nm, and 90 nm, respectively. When *θ* = 5°, EOF profile is not significantly affected due to less electric field distortion. As *θ* increases, the profile is more asymmetric as the average electric field along the nanostructured wall decreases.

For microchannel with parallel nanolines (i.e., *θ* = 0°), it has been deduced in our previous investigation [43] that there will not be any distortion of the external applied electric field distribution at the nanostructured wall because the nanolines are in the same direction as the applied electric field. EOF arises as a result of the interaction between the external applied electric field and the EDL. Thus, microchannel with nanolines parallel to the EOF flow has the same flow velocity as the smooth microchannel (without nanolines); this was verified experimentally in our previous investigation [43]. In this current investigation, it has been demonstrated through numerical simulations that the distortion of the applied electric field distribution is indeed less severe when the nanolines are almost parallel to the EOF flow (i.e., *θ* = 5°, which is near 0°) (see Figure 3). As such, the EOF velocity profile for microchannel with nanolines at *θ* = 5° is not significantly affected (see Figure 4), and the average EOF velocity approaches that of the smooth microchannel as expected (see Figure 2). 

When *θ* increases from 5° to 90° (i.e., nanolines perpendicular to the EOF flow), the simulation results revealed that the average EOF velocity decreases exponentially (see Figure 2). As reported by Kang and Suh [32] and Masilamani et al. [33], when the nanolines are perpendicular to the EOF flow, the distortion of the external applied electric field distribution has a strong dependency on the nanoline period *P* that dictates the nanostructure density on the microchannel surface. Our investigation shows that the apparent period *P_ap_*, as well as the apparent width *W_ap_*, of the nanolines (along the EOF flow direction) is a decreasing function of *θ* (see Figure 1 of Section 2.1). As *θ* increases, distortion that causes nonuniformity of the external applied electric field distribution within the vicinity of the nanostructured wall can be expected to increase due to the increased number of apparent nanolines per unit microchannel length, which is illustrated in Figure 3. Therefore, with increasing *θ*, the average electric field along the nanostructured wall decreases. This causes the EOF velocity profile to become more asymmetric (see Figure 4); this explains the overall EOF velocity reduction observed in Figure 2. 

**Figure 5:**
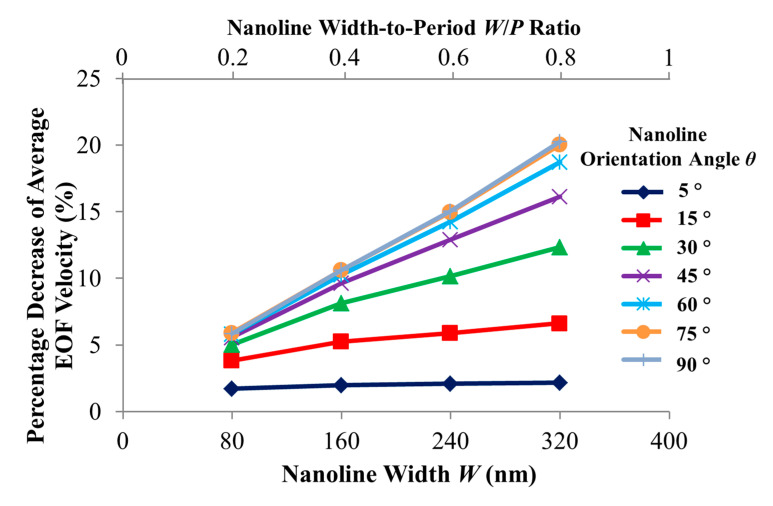
Percentage decrease of simulated average electroosmotic flow (EOF) velocity as a function of nanoline width *W* and orientation angle *θ*, as compared to smooth microchannel. Sodium bicarbonate (NaHCO_3_) concentration was fixed at 1.0 mM, and nanoline period *P* and depth *D* were fixed at 400 nm and 90 nm, respectively.

**Figure 6:**
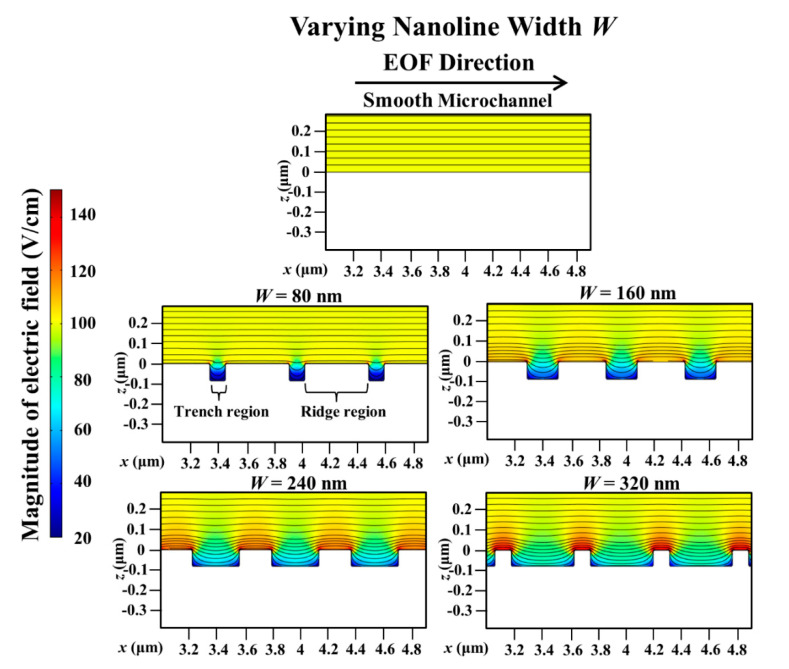
Simulated electric field distribution in microchannel with varying nanoline width *W*, in comparison to smooth microchannel. Sodium bicarbonate (NaHCO_3_) concentration was fixed at 1.0 mM, and nanoline orientation angle *θ*, period *P*, and depth *D* were fixed at 45°, 400 nm, and 90 nm, respectively. As *W* increases, the distortion in trench subsides leading to stronger field strength.

**Figure 7:**
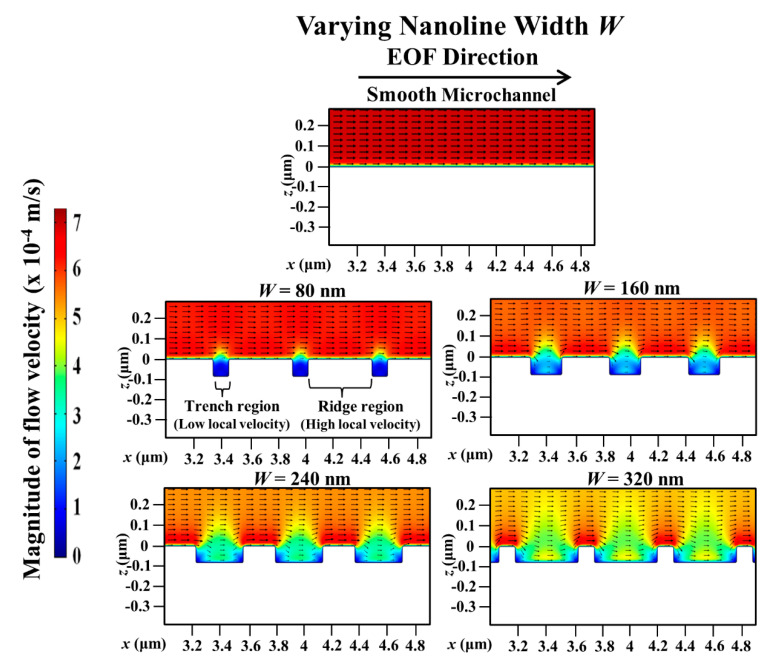
Simulated electroosmotic flow (EOF) velocity profile in microchannel with varying nanoline width *W*, in comparison to smooth microchannel. Sodium bicarbonate (NaHCO_3_) concentration was fixed at 1.0 mM, and nanoline orientation angle *θ*, period *P*, and depth *D* were fixed at 45°, 400 nm, and 90 nm, respectively. As *W* increases, the narrowing of ridge causes flow reduction and more asymmetric EOF profile.

With a smooth microchannel as the reference, Figure 5 shows the variation of the percentage decrease of the simulated average EOF velocity with the nanoline width *W* at various orientation angles. The decrease of the average EOF velocity is rather linear with increasing *W* for different *θ* (see Figure 5). Figure 6 indicates that the distortion of the external applied electric field distribution within the trench region subsides as *W* increases. As a result, the applied electric field strength along the nanostructured wall becomes stronger when *W* increases (see Figure 6); intuitively, this would accelerate fluid flow within and over the trench region (where low local fluid velocity is observed, see Figure 7). However, as *W* increases, the simultaneous narrowing of the ridge region (where high local fluid velocity is observed, see Figure 7) causes a retardation of the fluid flow velocity. Hence, when *W* is increased, the resultant flow reduction near the vicinity of the nanostructured wall surface leads to a more asymmetric EOF velocity profile (see Figure 7), and thereby an average EOF velocity reduction in the microchannel (see Figure 5).

**Figure 8:**
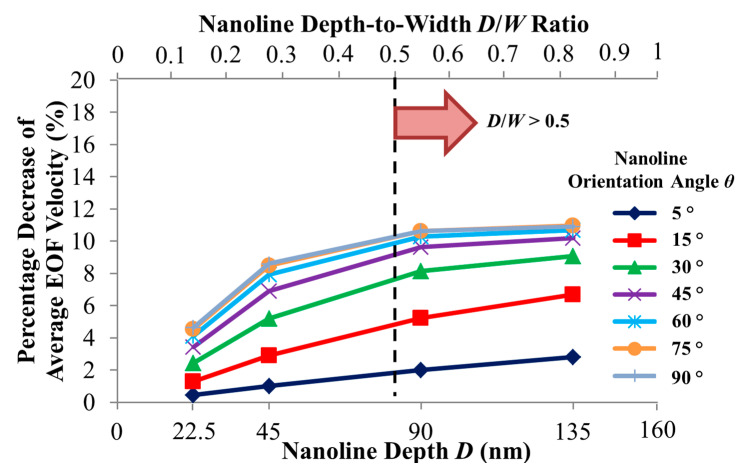
Percentage decrease of simulated average electroosmotic flow (EOF) velocity as a function of nanoline depth *D* and orientation angle *θ*, as compared to smooth microchannel. Sodium bicarbonate (NaHCO_3_) concentration was fixed at 1.0 mM, and nanoline width *W* and period *P* were fixed at 160 nm and 400 nm, respectively. Our investigation agrees with Kang and Suh [32] that EOF velocity reduction stabilizes when *D*/*W* > 0.5.

**Figure 9:**
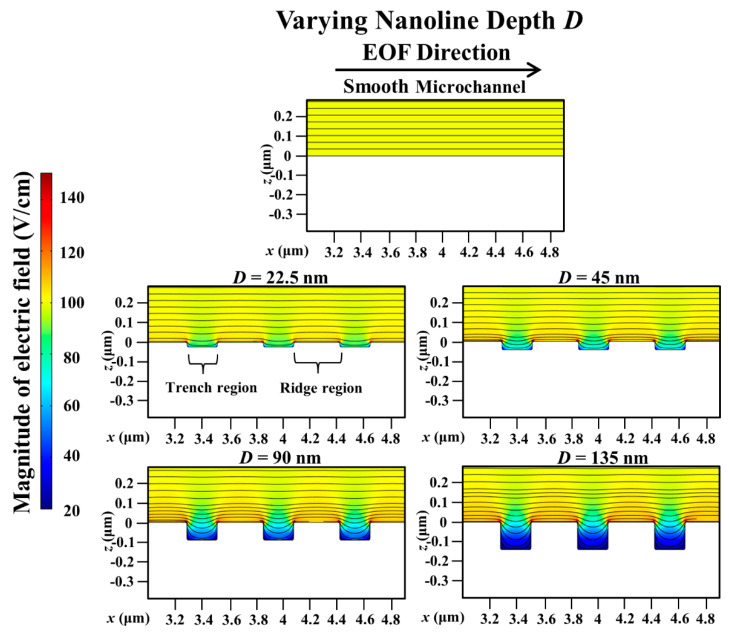
Simulated electric field distribution in microchannel with varying nanoline depth *D*, in comparison to smooth microchannel. Sodium bicarbonate (NaHCO_3_) concentration was fixed at 1.0 mM, and nanoline orientation angle *θ*, width *W*, and period *P* were fixed at 45°, 160 nm, and 400 nm, respectively. When *D* increases, there is more distortion of electric field in the trench. As *D* gets deeper, distortion within the trench remains nearly constant.

**Figure 10:**
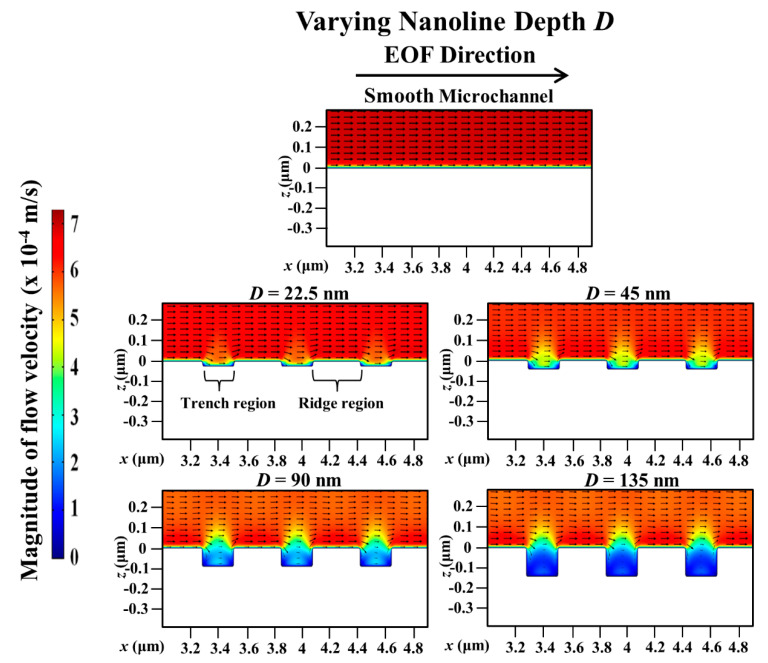
Simulated electroosmotic flow (EOF) velocity profile in microchannel with varying nanoline depth *D*, in comparison to smooth microchannel. Sodium bicarbonate (NaHCO_3_) concentration was fixed at 1.0 mM, and nanoline orientation angle *θ*, width *W*, and period *P* were fixed at 45°, 160 nm, and 400 nm, respectively. When *D* increases, EOF profile is more asymmetric due to more electric field distortion in the trench. As *D* gets deeper, EOF profile changes insignificantly as the electric field stays negligibly weak in the trench for driving EOF.

Compared with a smooth microchannel, the variation of the percentage decrease of the simulated average EOF velocity with the nanoline depth *D* for different orientation angles are presented in Figure 8. Figure 8 shows a monotonic increase of the percentage reduction of the EOF velocity with increasing *D* at various *θ*. The reduction in the EOF velocity is more pronounced when *D* ≤ 90 nm, and plateaus when *D* > 90 nm (see Figure 8). When *D* increases, there will be more distortion of the external applied electric field distribution inside the trench region (see Figure 9). Consequently, the applied electric field strength will not be sufficiently strong (see Figure 9) to drive fluid flow over the trench region (see Figure 10). Thus, with increasing *D*, the EOF velocity profile becomes more asymmetric (see Figure 10), and a decrease in the average EOF velocity is expected (see Figure 8). As *D* gets deeper (i.e., *D* ≥ 90 nm), the distortion of the applied electric field distribution within the trench region remains nearly constant (see Figure 9), and the electric field strength stays negligibly weak for driving EOF (see Figure 10). Indeed, Kang and Suh [32] had reported that the external electric field distribution hardly changes for nanoline depth-to-width aspect ratio *D*/*W* > 0.5, which results in insignificant changes of the EOF velocity beyond this aspect ratio. Similarly, our investigation demonstrates that when *D*/*W* > 0.5, the distortion of the applied electric field distribution has plateaued and the reduction of the EOF velocity stabilizes (see Figure 8). 

## 4. Conclusions

This investigation provides a thorough numerical analysis of the effect of varying the nanoline orientation angle *θ* on EOF. The percentage reduction of the average EOF velocity increases with an increase in *θ* for different concentrations of NaHCO_3_. This occurs as a result of the decrease in the average electric field due to the distortion of the local electric field distribution at the microchannel wall surface, with the presence of nanolines at various *θ*.

When the nanolines are almost parallel to the EOF flow (i.e., *θ* = 5°, which is near 0°), the distortion of the applied electric field distribution is not drastic. Thus, the EOF velocity profile for microchannel with nanolines at *θ* = 5° is not significantly affected, and the average EOF velocity approaches that of the smooth channel. As *θ* increases from 5° to 90° (i.e., nanolines perpendicular to the EOF flow), the average EOF velocity decreases exponentially. When *θ* increases, the applied electric field distribution distortion increases due to the increased number of apparent nanolines per unit channel length. Therefore, the average electric field along the nanostructured wall decreases. This causes the EOF velocity profile to become more asymmetric, which explains the overall EOF velocity reduction.

The effects of varying both the nanoline width *W* and depth *D* have also been investigated in conjunction with a variation of *θ*. Compared with a smooth microchannel, the percentage reduction of the average EOF velocity increases rather linearly with increasing *W* for different *θ*. The applied electric field distribution distortion within the trench region (with low local fluid velocity) subsides as *W* increases, leading to stronger electric field strength which intuitively should accelerate fluid flow over the region. However, the simultaneous narrowing of the ridge region (with high local fluid velocity) causes the retardation of the fluid flow velocity.

Increasing *D* at various *θ* shows a monotonic increase of the percentage reduction of the average EOF velocity. When *D* increases, there will be more distortion of the applied electric field distribution inside the trench region. Consequently, the electric field strength will not be sufficiently strong to drive fluid flow. As *D* gets deeper, the applied electric field distribution distortion within the trench region remains nearly constant; this results in the plateauing of the reduction of the average EOF velocity for nanoline depth-to-width aspect ratio *D*/*W* > 0.5.

This investigation reveals that the effects on EOF of nanolines, and by extrapolation of any nanostructures, may be directly attributed to their effects on the distortion of the applied electric field distribution within a microchannel.

## Figures and Tables

**Table 1:** Boundary conditions for 2-D simulation of a steady-state electroosmotic flow (EOF).

Variable	Condition ^a^	Boundary
Applied potential *φ*	*φ* = 0.28 V	Inlet
	*φ* = 0 V	Outlet
	−**n**·*σ*∇*φ* = 0	Smooth and nanostructured surfaces
Electrostatic wall potential *ψ*	**n**·∇*ψ* = 0	Inlet and outlet
	**n**·∇*ψ* = *S*/*ɛ_r_ɛ_o_* = (−9.86 × 10^−3^)/*ɛ_r_ɛ_o_*	Smooth and nanostructured surfaces
Concentrations of ions *c_i_*	*c_i_* = *c_o(i)_* exp(-*z_i_eψ*/*k_b_T*)	Inlet and outlet
	*−***n**·[*-D_i_∇c_i_ – u_m(i)_c_i_*∇*(φ* + *ψ)* + *vc_i_*] = 0	Smooth and nanostructured surfaces
Flow velocity *v* and Pressure *p*	*v* = 0	Smooth and nanostructured surfaces
	*p* = 0	Inlet and outlet

^a^ where **n** is unit vector normal to boundary, *σ* is solution conductivity, *S* is surface charge density, *ε_r_* is relative permittivity of fluid, *ε_o_* is permittivity of free space, *c_o(i)_* is bulk ion concentration, *z_i_* is ion charge, *e* is electron charge, *k_b_* is Boltzmann constant, *T* is temperature, *D_i_* is diffusion coefficient, and *u_m(i)_* is mobility of ionic species.

**Table 2:** Symbols and values of constants for numerical simulations, where ionic mobility of ion species is calculated by formula (*z_i_D_i_F*)/(*RT*).

Parameters	Symbol (Unit)	Value
Permittivity of free space	*ɛ_o_* (C·V^−1^·m^−1^)	8.85 × 10^−12^
Relative permittivity	*ɛ_r_*	80
Viscosity of water	*µ* (kg·m^−1^·s^−1^)	8.90 × 10^−4^
Density of water	*ρ* (kg·m^−3^)	1000
Faraday constant	*F* (C·mol^−1^)	96485
Gas constant	*R* (J·mol^−1^·K^−1^)	8.314
Boltzmann constant	*k_b_* (m^2^·kg·s^−2^·K^−1^)	1.381 × 10^−23^
Temperature	*T* (K)	298
Electron charge	*e* (C)	1.602 × 10^−19^
Avogadro constant	*N_a_* (mol^−1^)	6.022 × 10^23^
Diffusion coefficient of Na^+^	*D_Na_^+^* (m^2^·s^−1^)	1.334 × 10^−9^ [50]
Diffusion coefficient of HCO_3_^-^	*D_HCO3_^-^* (m^2^·s^−1^)	1.105 × 10^−9^ [50]
Ionic mobility of Na^+^	*u_m(Na_^+^_)_* (m^2^·V^−1^·s^−1^)	5.194 × 10^−8^
Ionic mobility of HCO_3_^-^	*u_m(HCO3_^-^_)_* (m^2^·V^−1^·s^−1^)	−7.919 × 10^−8^
Ionic charge number of Na^+^	*z_Na_^+^*	+1
Ionic charge number of HCO_3_^-^	*z_HCO3_^-^*	−1
